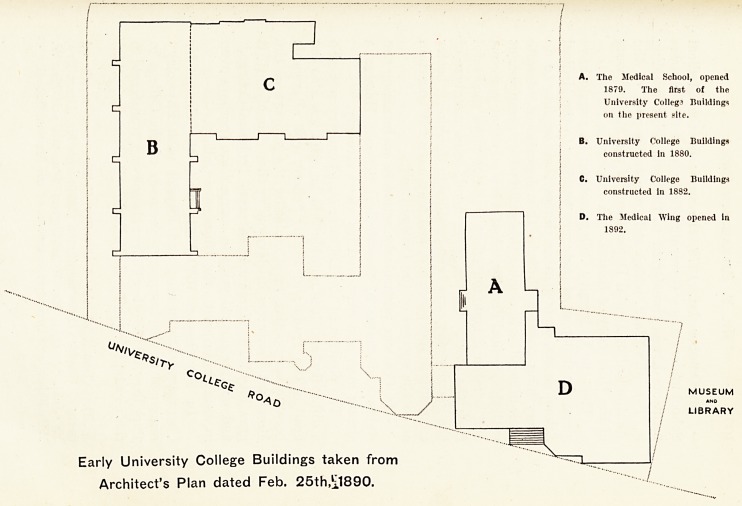# Early Medical Teaching in Bristol

**Published:** 1927

**Authors:** F. Richardson Cross


### The Bristol Medico-Chirurgical Journal

"*Scire est nescire, nisi id me
Scire alius sciret.*"

SUMMER, 1927.

INDEX

### EARLY MEDICAL TEACHING IN BRISTOL.

### THE BRISTOL MEDICAL SCHOOL AND ITS ASSOCIATION WITH THE UNIVERSITY COLLEGE.

BY

F. Richardson Cross, M.B. Lond., LL.D. Bristol, F.R.C.S.

On December 3rd, 1926, in celebration of the Fiftieth Anniversary of the foundation of University College, Bristol, a Reception for Alumni and friends was held at the University by the Vice-Chancellor and Professor G. H. Leonard, President of the Association of Alumni.

Addresses were given by the Vice-Chancellor (Thomas Loveday, Esq.), The Master of Balliol College, Oxford (Dr. A. D. Lindsay), Emeritus Professor C. Lloyd Morgan, F.R.S., the Principal Officer of the University of London (Dr. T. Sibly), Dr. H. S. Hele-Shaw, F.R.S., and by myself, as representing the medical side.

I have here put together some records and memories on which my remarks were based, as it seems worth while to print them as likely to be of interest and for future reference.

I date the real effort to deal with Medical Education in Bristol from the foundation of the Infirmary in 1735. For many years individual members of the Staff and others gave lectures somewhat irregularly, or courses of lectures, more or less associated, until in 1833 a group of teachers bound themselves together to form a Medical School.

In a building in Tyndall's Old Park Hill this school carried on useful work for forty-three years, and gave the necessary certificates of attendance on medical courses which were required by the examining bodies at that time.

The foundation, in 1876, of the University College of Bristol, with which the Medical School was associated, commences a new period in its history, as well as of higher education in Bristol. There was much progress in the medical sciences, and greater responsibilities were thrown on the teachers; but the improved buildings and equipment provided in the College gave the opportunity for better teaching and for more satisfactory study.

The work of the two bodies became more closely allied; until in 1893 they were incorporated, and so later the Medical School became the Medical Faculty of the University of Bristol.

In 1735, on September 22nd, a subscription was started for the purpose of founding an Infirmary for the sick poor of Bristol. The building was opened for patients on December 13th, 1737, and may thus lay claim, perhaps, to being the first hospital founded in the provinces. Four Physicians and two Surgeons had been elected on May 20th, 1737.

The two first Surgeons were William Thornhill, capable and skilful, a man of independent ideas and good private means, and Thomas Page. His son, John Page, was appointed in 1741, and James Ford was appointed in 1743. These two, after studying in Bristol, had both been in a London hospital and at the Hôtel Dieu in Paris. In 1746 they commenced a course of lectures on Anatomy, illustrated by dissections from the Infirmary "dead house," and possibly from other sources.[1]

Godfrey Lowe was appointed to the Infirmary in 1775. He gave a series of anatomical lectures and demonstrations, illustrated by dissections. Some of his "subjects" were sent to him from London.

Richard Smith, senior, was educated at Winchester College and afterwards as a student in London. He was a man of great ability and industry—Surgeon to the Infirmary, 1774 to 1791. He was a great collector of specimens, particularly anatomical and surgical, and of historical records. He founded the Infirmary Museum, which was said to have been the best of its type of that day, and he bequeathed his museum and relics to his son.

Richard Smith, junior, 1796–1843, was, like his father, a great surgeon and very interested in the Infirmary. He did much to extend the Museum, as he was an ardent collector of morbid specimens—especially those associated with murders—and he left a large number of medical works, some portraits, and the whole of his private museum to the Infirmary.

He died suddenly on January 24th, 1843, in the Philosophic Institute (now the Freemasons' Hall), and was buried in Temple Church. It was he who rescued from a lumber room at the Infirmary many important documents and papers dealing with the work of the Institution in past years. He collected these, with many cuttings from newspapers and innumerable other records, and bound them together in bulky volumes of Biographical Memoirs, which are still housed in the Infirmary. It is from these sources that writers on the early history of the Infirmary and of Bristol at that time (Latimer and others) have gained their information.

[1] "Early History of the Bristol Royal Infirmary," by J. Paul Bush, *Bris. Med.-Chir. J.*, xxvi.

From these also, and many other sources, George Munro Smith, Surgeon 1889–1909, wrote his valuable and fascinating book on the *History of the Bristol Royal Infirmary.*[1] Many of my comments in this pamphlet are condensed readjustments from Munro Smith's book.

The Infirmary became the local centre of a new impulse in medical science. It gave the opportunity to its doctors for meeting to investigate cases, to compare notes, to stimulate fresh ideas, and to suggest new lines for diagnosis and treatment.

Francis Bowles, the son of a barrister, of independent means, who lived at "The Fort," St. Michael's, was indentured as a pupil to Richard Smith, senior, in 1784, and he continued his education at Guy's and St. Thomas's. He returned to Bristol in 1790, and in 1793 was giving independent lectures on Anatomy.

In that year Dr. Thomas Beddoes, M.D., Reader in Chemistry, Oxon., came to Clifton, where he opened his "Pneumatic Institute" and the "Preventive Institution" in 6 Dowry Square. Humphry Davy was working as superintendent in his laboratory, testing the properties of nitrous oxide gas, which had been discovered by Priestley in 1776, but was not introduced into surgical practice until 1868. Beddoes, though eccentric, was a man of rare talent. He married Ann Edgeworth, and his home was the centre of an intellectual and literary group in Clifton which included Coleridge and Southey. An influential body of leading men of the day, attracted by his teaching, begged that he would arrange for a course of scientific lectures, particularly in Anatomy, a very mysterious and forbidden subject at that time.

Bowles appears to have been a very gifted man—an excellent anatomist, a great reader, a good linguist, but young, and thoroughly enjoying the pleasures of life. At the same time he was a very hard worker, and a strong advocate for proper anatomical teaching. He

[1] Arrowsmith, Bristol, 1917.

gave practical anatomical instruction to the Infirmary students gratuitously, and also to members of the Staff. Dr. Beddoes invited Bowles to help him lecture, and Bowles requested that Richard Smith, junior, should be asked to join them, as he had a good museum with a large number of specimens. They issued a prospectus in 1797, hired a large room at the Red Lodge, Stoney Hill, and lectured on the "Structure of the Frame," and in 1798 on "The Senses." So successful were they that they decided to build a theatre for lecturing purposes, on Chemistry and Anatomy, inside the Archery, in Lower College Green. Bowles succeeded under great difficulties in getting subjects for dissection, which were essential for the teaching. He was appointed Surgeon to the Infirmary in 1806, but died in 1807, probably from phthisis.[1]

Thomas Shute was a pupil of Bowles and Richard Smith, junior, 1798–1799. He studied afterwards for three years at the London Hospital and in Edinburgh. He returned to Bristol in 1805, and in 1806 advertised his lectures on "Anatomy for the Medical Students of Bristol." He gave a second course in 1807 on the "Principles and Operations of Surgery." Shute built a lecture room, "The Anatomical Theatre," Lower College Street, 1808, at the back of his father's stables. He took great pains over his lectures, made good dissections on subjects he bought (some of them brought by wagon from London), and demonstrated and lectured upon them. This was the first start of proper anatomical instruction in Bristol. He held the office of Surgeon to the Infirmary from 1812 till 1816. He was a good dissector and lecturer, and very popular. He lectured only to medical students, who had to find their own subjects. His largest class was twenty-four.

At Shute's death in 1816 Dr. George Wallis, one of his pupils, took over his Theatre of Anatomy, giving

[1] Latimer's *Annals of the Eighteenth Century*, pp. 264, 524.

his first course there in 1817. He also lectured on Comparative Anatomy at the Bristol Institution. He was a very keen anatomist, and had, no doubt, personally assisted in obtaining bodies for dissection.

James Cowles Prichard, 1786, studied at St. Thomas's Hospital and Edinburgh, where the subject of his thesis for the M.D. was "de Generis Humani Varietate." He afterwards went to Cambridge and to Oxford. He came to Bristol in 1810, and in 1814 lectured at his own house on the "Physiology, Pathology and Practice of Physic." He and Dr. T. E. Stock (Physicians to the Infirmary, 1811–1828) asked leave of the Committee to give lectures on Medicine in the Infirmary, illustrated by cases in the wards, advancing the argument, "We are the more disposed to prefer this request because it is an object of ambition to attempt to lay the foundation of a Medical School in an institution which furnishes so may valuable opportunities for professional improvement." The lectures were given in 1816. J. C. Prichard was Physician to the Infirmary, 1816–1843. He was a great investigator and student of many subjects. In 1819 he wrote a book on Egyptian Mythology. The work of his thesis for the Edinburgh degree was much widened and elaborated, until in 1831 he published his great book on *Researches into the Physical History of Man,* which gained him his F.R.S. and an honorary M.D. In 1845 he was made one of the Commissioners in Lunacy, London.[1]

Courses of lectures were being given in Bristol, particularly at the Philosophical Institution, the City Library Literary Scientific Institute, on general knowledge, and some on scientific subjects related to Biology and Medicine. The courses included demonstrations on Anatomy, a subject which aroused wide interest among the public. Many of the younger surgeons made

[1] See also Henry Alford, "Student Days," *Bris. Med.-Chir. J.* viii., 176.

dissections and gave lectures on Anatomy before they were appointed on the Infirmary Staff.

To teach Anatomy it was essential to get subjects; the teachers were licensed, also the lecture rooms. The bodies of murderers had been handed over to the Surgeons of the Infirmary for dissection for some years, other bodies or parts could be dissected legally, and other subjects were acquired in less legitimate ways.[1]

William Hetling was Surgeon to the Infirmary 1807–1837. On March 9th, 1814, he wrote to the Treasurer asking permission to give a course of lectures on "The Principles and Practice of Surgery," in some suitable rooms at the Infirmary. He proposed lecturing twice a week to the students, or to any scientific gentlemen who might wish to attend. The request was granted, and the lectures were advertised. His fellow-surgeons, however, raised difficulties, and it was not until February, 1831, that with the full approval of the Staff he gave lectures on Surgery in the Infirmary Museum. They were freely advertised and led to wide correspondence in the Press. A full syllabus was published in 1833. In his first lecture to about fifty people he drew attention to the excellence of the museum and fine collection made by Richard Smith. He said, "This highly-gifted individual naturally attracted the attention of myself and the rest of the pupils of that day towards him, and on one distinguished occasion he most kindly and considerately invited me to his house to meet the present Sir Everard Home, who came to Bristol to view his museum. At that period, after going over every individual specimen, which occupied several hours, I perfectly recollect this gentleman's declaration that it was the most unique, skilful and extensive museum he had inspected as the

[1] See W. H. Harsant, "Medical Bristol in the Eighteenth Century," *Bris. Med.-Chir. J.*, xvii., and Munro Smith, *History of Bristol Royal Infirmary*, 1917, chap. xvii.

production of one individual, excepting of course the unrivalled Hunterian Collection."[1]

The many treasures contained in Richard Smith's museum, which was especially rich in calculi from the bladder and examples of bone diseases, had been carefully preserved, and other pathological specimens had been added from time to time.

In 1860 a new Museum was built at the same time as the Infirmary Chapel, and all the specimens re-arranged. It was publicly opened by Dr. William Budd.

Recently a new laboratory has been added above the Museum, by the generosity of the employees of Christr. Thomas and Bros., as a memorial to their fellows who made the supreme sacrifice in the Great War, 1914–1918.

The new department was planned and constructed under the advice of Dr. Alex. Fraser, the Curator of the Museum and Pathologist.

Also at the General Hospital there is a good Pathological Department and a well-arranged Museum and Laboratories. The building was erected by the generosity of the late Mr. Fenwick Richards. This department was considerably extended by him, and a well-equipped modern laboratory added, the whole department being under the direction of the Pathologist, Dr. G. Hadfield.

The University, too, possesses a first-rate Pathological Department with a well-appointed Museum, and laboratories for all kinds of investigation, under the control of the Professor, Dr. Walker Hall.

Dr. George Wallis gradually extended the work of Shute's School, adding lectures on Surgery, Midwifery, Anatomy, Physiology, Materia Medica. Dr. Riley, Dr. Howell (later both Physicians to the Infirmary) and Mr. Halse with others lectured there. It became known as the "School of Anatomy and Medicine."

Thus the independent lecturers were gradually associating themselves together in groups, and dealing with a wider range of subjects by means of a choice of lecturers each competent in his particular subject.

In 1826 Henry Clark[2] opened a fresh "Theatre of

[1] Munro Smith, *History of Bristol Royal Infirmary,* p. 373.

[2] *Ibid.,* p. 309.

Anatomy " at the back of King Square, in competition with Wallis's School. In 1827 he enlarged his lecture room and was joined by William Herapath, 1828, who then gave the first course of lectures in Chemistry recognised by the Apothecaries' Hall. In 1829 the Anatomical Theatre was entirely rebuilt, the dissecting room much improved, and other lecturers were added: Dr. Chadwick, Nat Smith, T. B. Taylor and Mr. Rootsey. In 1833 Dr. Riley, who had been lecturing at Wallis's School, joined them, and added much to their claim to be a complete Medical School. There were forty-nine pupils in Anatomy and between thirty and forty in Chemistry. This institution was now called the "Bristol Medical and Surgical School," and received recognition from the College of Surgeons and the Apothecaries' Hall.

Meanwhile Shute's, the old "School of Anatomy and Medicine," continued under Dr. Riley, Dr. Wallis, Dr. Davies and Dr. Halse.

The need for proper training of students in Anatomy, particularly, as well as in all subjects bearing on the treatment of disease, was being more fully recognised, not only by the medical profession, but by the public at large. The Staff of the Infirmary were taking an increasing interest in the value of the courses of lectures, which were being given more or less independently of them. The passing of the Anatomy Act in 1832 allowed bodies to be procured for dissection and demonstration in authorised licensed places.

Meetings were held in 1833, and inquiries made to try to secure co-ordination of teaching by uniting these two Anatomical Schools, and thus forming an extensive basis upon which lectures in the other departments of medical education could be added, so as to form a complete and effective Bristol School of Medicine. Meetings between Mr. H. Clark (Anatomy), Dr. Riley (Anatomy), William Hetling (Lecturer on Surgery at the Infirmary), William Herapath (Chemistry), were held in the month of August, 1833, with a view to effect such junction. It was resolved to issue a prospectus for a new school, 1833–1834. Dr. Riley decided to leave the "School of Anatomy and Medicine" to associate himself with H. Clark, and to share the chair of Anatomy with him, while continuing to lecture also on Medicine. Their colleagues were William Hetling (Surgery), William Herapath (Chemistry), S. Rootsey (Botany). Dr. Wallis was unwilling to leave his colleagues Dr. Howell and others, on the "School of Anatomy and Medicine," though he was repeatedly invited to do so; he consented and changed his mind several times.

The Bristol General Hospital had been founded in 1831, and from its staff Mr. G. D. Fripp was offered the lectureship on Materia Medica, and Dr. J. A. Symonds that of Forensic Medicine; while Mr. J. C. Swayne, of the Dispensary, was elected to lecture on Midwifery. The above eight constituted the staff of the new school. The list of the lecturers was published in the first "Prospectus of the Bristol Medical School for the Session 1833–34," and will be found in *The Bristol Medico-Chirurgical Journal*, vol. x., p. 266, in a valuable and very interesting paper by Mr. Augustin Prichard on the early history of the School and of its later progress.

Mr. Augustin Prichard was perhaps the most distinguished of our older teachers and surgeons; he did noble work for the Medical School, and for surgery and ophthalmology in the West of England. He died December, 1898.[1]

From 1832–33 onward my notes have been taken from the three volumes of minute books which were kept by the lecturers at the old Medical School in the Royal Fort, from 1833 to 1876, and now in the care of Mr. Geoffrey Francis, Registrar of the University of Bristol. The minutes of the Faculty meetings were full

[1] See *Bristol Medico-Chirurgical Journal*, xvi., and *A Few Medical and Surgical Reminiscences*, by A. Prichard (Arrowsmith, 1896).

and carefully taken, and give the history of the School for that period.

Dr. Andrew Carrick, the Senior Physician at the Bristol Royal Infirmary, gave the inaugural address on the opening of the School, October 14th, 1833. Commenting on the importance of proper and full medical education and the advantages to be gained for the community by a School of Medicine in Bristol, he added, "This is the first time that an adequate number of reliable and talented individuals have been united here for the purpose of giving instruction in every department of medical science. The first time that anything has appeared here which could have any title to the character of a Medical School."

He pointed out the great advantage to those living near Bristol to be able to educate their sons fully for the profession whilst having the advantages of home life, and also how the possession of such a school would lead to a higher standard of medical knowledge in its midst. Moreover, he considered it very desirable that a school of medicine should form an integral part of the Bristol College.

On November 28th, 1833, at a meeting of the Lecturers at 15 Park Street, a sub-committee of the Faculty was appointed to confer with the Council of the Bristol College in Park Row as to the possibility of union between the two institutions and of obtaining lecture rooms in the College for the new Medical School.

After some consultations and correspondence with the Council and with Dr. Jerrard, the Principal, no arrangements could be arrived at.

Dr. J. G. Swayne, in the Obituary Notice to Augustin Prichard,[1] gives an interesting account of this College, which was opened in 1831 by some of the leading citizens of that day, aided by members of well-known medical families, in a fine old house in Park Row, opposite to the Red Lodge. In it were educated the Swaynes, the Prichards, two of whom gained high distinctions at Oxford, and Samuel W. Wait, afterwards a Don at Trinity, who later served on the Bristol Infirmary Committee, and others. Despite its very high record of successes, it only lasted eleven years, and was followed by the Bishop's College.

On January 6th, 1834, a Parliamentary Committee had been appointed to inquire into the state of medical education in the

[1] *Bris. Med.-Chir. J.*, xvi., 1.

country. At that time a six months' surgical course was required at a London hospital by the College of Surgeons, and they were asked that this practical work should be as fully recognised in the Bristol Infirmary as in a Metropolitan hospital. In August, 1834, the Infirmary was registered as equivalent, for this purpose, to the Metropolitan hospitals.

On April 16th, 1834, a meeting of the associated Lecturers took place in premises at the bottom of Old Park Hill, formerly used as a school by Dr. De Boudry, and adjoining the Certified Industrial School. These premises were obtained on a lease from Mr. Edward Clarke. An Agreement of Association among the Lecturers of the Medical School was drawn up and signed in June, 1834, stating "That we hold ourselves bound for the joint maintenance and conduct of the School of Medicine, Surgery and Anatomy."

On October 20th, 1834, Mr. Richard Smith, junior, gave the second introductory address. Meanwhile the management of the School was entirely in the hands of the Lecturers, who practically owned it, and did as they pleased. Various shiftings were made among the Lecturers from time to time.

On September 1st, 1835, James F. Bernard, M.B., was appointed Lecturer on Materia Medica, but a correspondence followed, the Apothecaries' Hall declining to acknowledge his lectures until he became M.D., or else a licentiate of the Royal College of Physicians of London. Dr. Bernard qualified by taking a Cambridge M.D. In October he signed the Agreement of Association, the first to do so after the original list of eight members.

On February 27th, 1836, Dr. Riley, anxious to lessen any opposition to the new School, offered to resign his Lectureship on Medicine to Dr. Wallis, who wished to join on public grounds, but found difficulty in arranging with his present colleagues in Shute's School, who did not wish to free him, although Dr. Howell (Physician, Royal Infirmary, 1829–1843) raised no objection. The question of Dr. Wallis's joining stood unsettled for a long time. He appears to have given one or two lectures or courses of lectures more or less independently till 1836. He was Physician to the Infirmary, 1828–1855.

On September 1st, 1836, a suggestion for a dinner for lecturers with students was made, Dr. Carrick to preside. Thirty-five students gave their names, but an outbreak of influenza stopped it.

On November 16th, 1838, a discussion is recorded as to the steps which it might be desirable to take with reference to the new University of London, and the propriety of establishing additional courses of instruction in Physics, Geology, etc., and further, on May 13th, 1839, Physiology, General and Comparative Anatomy, General Pathology, Pathological Anatomy, Hygiene. In July, 1839, a letter from the Registrar of the University of London recognised this School.

On January 6th, 1840, W. B. Carpenter was appointed to part Chair of Anatomy, and also Secretary of the School, vice G. D. Fripp.

In August, 1841, Augustin Prichard was appointed Demonstrator of Anatomy, and Secretary in 1844. He made many valuable cuttings in regard to the School, which are found in one of the minute books alluded to.

On April 26th, 1845, the prizes at the Medical School were distributed by Sir J. K. Haberfield. The *Bristol Mirror* made this comment: "We think the Faculty of the School have exercised a wise discretion, when the important subject of medical education occupies so much of public attention, in deciding on a public distribution of prizes. The advantages of a local institution, in which young men destined for the profession may pursue their studies without leaving home, can hardly be appreciated too highly by their parents and friends."

In the course of this address Sir J. K. Haberfield said:—

"The honour of establishing the first regular school for teaching Anatomy in the provinces belongs to Mr. Thomas Shute, who began his lectures in 1805, and continued them uniformly in regular courses every year until his death in 1816, when Dr. Wallis, as has been recorded, undertook the arduous duties of continuing the School, which he maintained for upwards of twenty years. In 1818 the Royal College of Surgeons and the Apothecaries' Society transmitted to Dr. Wallis their official recognition of his School. Be it remembered that this was the first School acknowledged by those public authorities out of London. It was the citizens of Bristol who made the first advance, and first broke through the great monopoly in medical education which had since the abolition of the Guilds of Barber-Surgeons been solely in the hands of the Metropolitan profession.

Manchester was the next School (in 1818), then Liverpool and Birmingham. The School from which we may claim descent was commenced by Mr. Henry Clark in 1828."

The buildings were, however, not occupied until 1833. They were so hidden away in the bottom of Old Park Hill, at the end of "Medical Avenue," that a stranger coming to Bristol would have had some trouble to find out where they were situated. I recently went over the premises with Colonel Paul Bush, who was a student here. The yard that is reached by a double gateway and leads to a large front door we identified. The front door opened into a small hall, on the right side of which was Fitzpatrick's room, where he injected the subjects and prepared them for dissection. Above this room was a double dissecting room, on the left of the hall was a lecture room used for chemistry, and beyond this a chemical museum. A staircase led up from the hall directly into the larger central lecture room, and by this door the students reached their seats. On the left side of the staircase was a room used by the lecturer and prosectors for making dissections for the lecture, and it had its own entrance into the lecture theatre, through which the lecturer entered. On the right side of the staircase was the students' dissecting room, and beyond the large lecture room a good-sized museum and gallery.

I remember these rooms and the old School, when for a few months, just after passing my London matriculation, I was attending lectures on Chemistry here under Mr. Herapath, and at the Trade School under Mr. Thomas Coomber, and on Botany under Mr. Leipner, and on Physics. I also lectured here from October, 1878, to October, 1879, for a year when I first came to Bristol.

In these humble quarters the Bristol Medical School with its devoted group of teachers did good work for forty-five years, and helped to provide students and dressers for the Bristol hospitals, barely paying their expenses from the fees, and without any other pecuniary aid, and with little thanks or recognition from the public or even the profession.

In 1860 special classes were adapted for the requirements of the Pharmaceutical Society, and in 1861 for dental students, as required by the College of Surgeons.

Mr. Henry Clark died on August 21st, 1861. He was associated with the School for upwards of thirty years.[1]

On August 9th, 1861, Mr. Coë took half the Chair of Surgery with Mr. Augustin Prichard.

On March 20th, 1863, it was resolved that the Chairs of Anatomy, Physiology, Surgery and Medicine should be equally divided by the appointment of an Infirmary and a Hospital representative to each Chair.

On July 5th, 1864, Mr. Leipner was appointed Lecturer on Botany.

The lease of the premises came to an end March 25th, 1867, and it was renewed for seven years, up to 1874.

On February 14th, 1868, occurred the death of Mr. Herapath, As his successor Thomas Coomber, F.C.S., was appointed.

In 1869 Dr. Long Fox joined Dr. Martyn in the Chair of Medicine.

On February 18th, 1870, a circular was received from the Faculty of Medicine, Manchester, suggesting the formation of an Association of Teachers of provincial schools ; and later one from Reginald Harrison, Liverpool, inviting us to a meeting at Newcastle.

On March 1st, 1872, a question arose as to renewal of lease. It was decided to try and find new premises, but without success.

On October 4th, 1872, Tibbits suggested a public movement to provide new buildings for the School of Medicine (as had been already done in Liverpool). The Faculty at its next meeting appointed a special committee to consider the advisability of appealing to the public to help to provide suitable premises.

On November 1st the questions of the appeal were considered. A sub-committee was appointed consisting of Mr. Tibbits, Mr. Atchley and Dr. Long Fox (Dr. Swayne was added

[1] See Dr. J. G. Swayne in *Brit. M. J.*, 1861, ii., 363.

on December 9th), and Dr. Burder as Secretary, to communicate with some influential citizens, as well as with the Committees of the Infirmary and Hospital, and general inquiries to be made.

On December 6th the question whether it was desirable to take measures to build a new School arose, and it was decided that it was. Also a site at the junction of Griffin Lane with new Colston Road, on which the City Surveyor was consulted, was examined, together with other places.

On January 24th, 1873, circulars were distributed, estimating the expense at £4,600, to the Faculty and doctors in the neighbourhood, and among the gentry in the city and suburbs. The scheme met with approval, and promises of support came from a number of them.

On February 7th, 1873, Mr. Coomber proposed that instead of proceeding with an independent scheme the School should seek co-operation with the authorities of the Museum and Library in a joint effort to establish a "College of Science of which the Medical School should be one department." It was resolved to propose a Conference. On February 21st, in reply to this, Mr. Lewis Fry, Hon. Secretary of the Museum Library, stated that five gentlemen had been appointed to meet the deputation from the Medical School, which consisted of Dr. Swayne, Mr. Coë, Mr. Coomber, Mr. Tibbits and Dr. Burder, the Secretary. Several meetings were held, and Mr. Coomber was to present a definite scheme. On March 21st an adjourned meeting of the deputation of the Museum and Library with the Medical School was reported, several other gentlemen being present by invitation. A decision that the Museum Library and the Medical School should each appoint delegates to form a Committee for the prosecution of the general scheme was approved. On October 3rd, at a Faculty meeting, Mr. Tibbits drew attention to the fact that matters relating to the proposed College of Science seemed to have been in abeyance. It was stated that the meetings which had been suspended would shortly be resumed.

It is convenient here to draw attention to the great interest that Dr. Percival, the Head Master of Clifton College, had for some time been taking in national education.

In February, 1868, Dr. and Mrs. Percival and some others had formed a Committee to promote the higher education of women. They had obtained the services of some very distinguished men as lecturers. Miss Catherine Winkworth became Secretary. Later, classes were started to aid women for the higher Cambridge examination. The lectures and classes were very successful, and gave an impulse to higher intellectual life in Clifton.

In 1872 Dr. Percival had written a pamphlet on the "Connection of the Universities with the Great Towns." He thought that there should be founded local colleges, but that in addition to suitable buildings and salaries for teachers provided by the local authorities the older Universities should take an interest in the matter, and certain of their colleges might find means of supplying teaching of the higher standards, or even possibly one or two professorships might be undertaken by them in the colleges of the larger cities. He did not wish the local colleges to be purely under local control and initiative, but that they should be directly linked up with the older University culture; while the professors would gain something by contact with civic life and with the commercial classes. Indeed, he had formulated the details for carrying this idea into effect.

This was the beginning of a new movement. He had gained some good support in Oxford, particularly from Professor Jowett of Balliol, and later from New College, but there was considerable hesitation felt as to allocating funds to be spent outside the University itself. Percival considered his ideas specially applicable to Bristol, and they were no doubt well appreciated by his Clifton friends.

When, therefore, meetings were held in Bristol suggesting the extension of the Medical School and its participation with the Museum and Library in a new College of Science, it was natural that Dr. Percival, and those who advocated the foundation of a local college for the higher education of adult men and women, should make a move. Accordingly, we are not surprised that at the meeting of the Bristol Medical School Faculty, March 6th, 1874, Mr. Coë reported the fact that negotiations were in progress between the Committee of the College of Science in Bristol and certain college authorities in Oxford with a view to joint action. In order to secure the co-operation of Oxford it would be necessary to get a preliminary guarantee of local support. On May 1st, 1874, the Secretary of the Faculty reported that at the Committee Meeting of the proposed College of Science the sum required for this guarantee had been over subscribed.

About this time a circular was issued proposing that a School of Science and Literature for the West of England and South Wales be established with the co-operation of certain colleges in the University of Oxford. It was signed by Gilbert Elliot, Dean of Bristol; W. Proctor Baker, W. B. Carpenter and Lewis Fry, Hon. Secretaries; and R. Shingleton Smith, M.D., Secretary.

On June 11th, 1874, a public meeting was called together[1] and held at the Victoria Rooms, Clifton, under the Presidency of the Mayor of Bristol, Mr. T. Barnes, who was supported by the Bishop of Exeter (Dr. Temple), Professor Williamson (President of the Royal Association), Rev. Professor Jowett (Master of Balliol), Rev. Dr. Sewell (Warden of New College), Dr. W. B. Carpenter (Registrar, London University), The Dean of Bristol (Dr. Elliot), Rev. Dr. Percival, E. A. Freeman, Dr. Roscoe (Owens College, Manchester), and many others.

[1] Latimer's *Annals of the Nineteenth Century*, p. 474.

The meeting was very successful, and the movement to establish a University College for Bristol and the West of England was well supported, and many donations were promised, including £1,000 on the part of the Medical School to the common fund, and the conditions under which the College and the School might be allied were carefully considered.

For some time the Bristol medical students had been doing badly at their primary examinations. The College of Surgeons was finding fault with their knowledge of Anatomy and Physiology. At the local hospitals the Staffs complained that the supply of dressers and clinical clerks was suffering (and later, moreover, on March 3rd, 1879, at the Faculty meeting, a letter was read from the Royal College of Physicians calling attention to the deficiency of knowledge shown by the candidates for the College licence in Chemistry as applied to Pharmacy and Toxicology, and in Pathology). It was evident that the teaching was not satisfactory, and that help and supervision were needed. The Infirmary members of the Faculty believed that a radical change was necessary, and that the proper remedy for better teaching would be a full incorporation of the Medical School with the proposed University College.

The Hospital and other members thought a less complete fusion would suffice, which should leave the Medical Faculty more independent, with its museum, and such property as it possessed, in its own hands. They considered it was necessary in the interests of medical education that the Faculty of the Medical School should retain its present status, but that future vacancies should be filled by a General Council.

On July 11th there was a meeting to consider the two alternative schemes of complete "incorporation" with the College of Science and Literature, or merely of "affiliation" of the two Schools.

On November 20th, 1874, affiliation was decided upon, and "that the Medical School should be managed by a Committee, consisting of its own Faculty—taking its fees and paying its own expenses, together with some help as to paying fees from the College ; but all vacancies hereafter to be filled by an Electoral body, with the exception of Chemistry and Botany." The two Medical Lecturers on these subjects were not to be given professorial rank, as it was intended that full chairs in these subjects would be filled independently by the University College Council. A minute on October 16th, 1874, showed that this was one of the main objections to "incorporation." The Faculty Book showed a note by Mr. Coë : "In addition to monetary concessions we have never felt comfortable in, as a body, allowing two of our number to make such sacrifices. In consequence of the discussion having arisen on the question of the Chairs of Botany and Chemistry, an opportunity has been given of amending an error into which we have fallen without due consideration. The matter having been re-opened, we feel it incumbent upon us to keep these two gentlemen associated with us at least in our own teaching."

On January 7th, 1875, Mr. R. W. Tibbits wrote a letter to the newspapers drawing attention to the fact that nearly two years previously a subscription had been suggested to build or provide a satisfactory Medical School for Bristol, such as had been provided in Liverpool by public subscription, and at Manchester as a part of Owens College. Other comments were made in the Press as to the immediate and urgent needs of the Medical School and the Technical College of Science.

On July 21st, 1875, there was an important meeting of peers and members of the House of Commons under the chairmanship of the Earl of Cork, for the purpose of considering the scheme for promoting the School of Science and Literature, which was proposed to be established in Bristol, and an article in the Press of July 22nd, 1875, headed: "Proposed New University for Bristol," said that "The affiliation of the Medical School with the Technical College is doubtless a commendable feature in the project. Two Oxford Colleges, New and Balliol, the former among the richest and the latter among the poorest of the list, have placed £300 a year each at the disposal of the New University for a time."

On October 1st, 1875, Dr. Shingleton Smith, who had been very active as Secretary of the new University College, was sent abroad for his health.

On November 5th, 1875, Mr. Coë reported that the new University College[1] proposed to buy a new site in Tyndall's

[1] Bristol University College was incorporated on August 9th, 1876.

Park, behind the Museum and Library, and to erect buildings there; or else temporarily to take what is now the Territorial Engineers' Headquarters in Park Row, and modify the existing buildings.

On February 4th, 1876, Mr. Coë was appointed as a representative of the Medical School on the Council of the University College, Mr. Tibbits on the Board of Governors, and Dr. Burder Governor also by the promise of £1,000.

On April 20th, 1876, a memorandum of agreement for affiliation of the Medical School and its new Electoral Body with the University College was agreed to.

The Electoral Body appointed for the Medical School consisted of the present members of the Faculty, including Mr. Coomber and Dr. Leipner (twelve in all), four representatives of the Royal Infirmary, four representatives of the General Hospital, and three representatives of the Council of the College. The Museum and other property to remain that of the Medical School. The Medical School to have representatives on the Board of Governors of the College, on the Council, and on the Educational Board.

### THE UNIVERSITY COLLEGE, BRISTOL.

The work of the University College began in the large building formerly known as Woodland House, in Park Row, October 10th, 1876. Examination for scholarships had been held on October 3rd.

### SYLLABUS, 1876–1877.[1]

#### COUNCIL.

*President*, Dean Elliot. Appointed by Governing Body of the College: *Chairman*, F. N. Budd, Esq.; *Vice-Chairman*, Right Hon. Lewis Fry; *Treasurer*, W. Proctor Baker, Esq.; Rev. Dr. J. W. Caldicott; Rev. F. N. Gotch; Rev. Dr. Percival; G. F. Schacht, Esq.; William Smith, Esq. Nominated by: Vice-Chancellor of the University of Oxford, Rev. B. Jowett, Balliol; Vice-Chancellor of the University of Cambridge, James Stuart, Professor of Mechanics; Vice-Chancellor of the University of London, W. Lant Carpenter; Bristol Medical School, R. W. Coë; New College, Oxford,

[1] Issued in 1877–1878, together with an abridged prospectus of the School of Medicine.

Hereford B. George; Balliol College, Henry J. H. Smith, Professor of Mathematics, Oxford.

TEACHING STAFF.

*Professors:* Chemistry, E. A. Letts, Gottingen; Modern History, James Rowley. *Lecturers:* Chemistry, W. W. Nicol (Demonstrator); Mathematics, W. R. Bousfield; Experimental Physics, Sylvanus Thompson; Zoology, Adolph Leipner; Botany, A. Leipner; Geology, E. B. Tawney; Classics, —; Political Economy, P. Hallett; French, —; Logic, —.

Feeling was still running very high between the members of the Medical School as to complete amalgamation or not with the University College. The Infirmary members very strongly advocated complete incorporation, and they at last decided to invite the College of Surgeons to investigate the position! They asked the other members of the Faculty to take this action with them, which was declined.

On June 9th, 1878, at a meeting of the Infirmary teachers it was decided to prepare a scheme by which an independent Infirmary school could be established, in regard to lecturers, premises, and funds. Many of the best-known older members of the Infirmary Staff agreed to resume lecturing, and it was decided to invite two or three new members to join them, and a satisfactory syllabus was provided. Dr. Spencer and Mr. Tibbits interviewed the President and Secretary of the Royal College of Surgeons, and a promise of recognition was given subject to certain conditions—among them that the teaching in Anatomy and Physiology should be reorganised and strengthened. This energetic action produced an altered attitude, and brought the other members of the Faculty to a compromise; and although incorporation was not agreed to, it was arranged that a combined "governing body" for the Medical School should be appointed, on which the Council of University College, the Committees and Staffs of the two Hospitals, and the lecturers of the Bristol Medical School should be represented.

I venture here to introduce a personal note. I was then working at King's College, where I was Sub-Dean and Medical Tutor and Lecturer on Physiology to the Evening Classes. I was approached by Dr. Shingleton Smith, an old King's College man and a personal friend, and asked by him if I would consider the prospects of coming to Bristol to lecture on Anatomy. I decided that if I could also be appointed Surgeon on the Infirmary Staff I would come.

Mr. Crosby Leonard, who was in poor health, resigned from the Infirmary on August 3rd, 1878. Mr. Arthur Prichard was appointed in his place.

I addressed an application for Mr. Prichard's vacancy from King's College Hospital, with testimonials to the Governors of the Infirmary, for they made the election in those days. I was elected Assistant Surgeon on September 10th, 1878, at a meeting of the Governors in the Board Room of the Infirmary, without opposition.

On September 18th, 1878, the newly-constituted Electoral Body for the Medical School made the following appointments (thirteen members being present): Lecturers on Operative and Practical Surgery, Mr. W. P. Keall and Mr. Arthur Prichard; sole Lecturer on Physiology, Dr. Shingleton Smith; sole Lecturer on Practical Physiology, Dr. G. F. Atchley; sole Lecturer on Anatomy, Mr. F. R. Cross.

We began lecturing in the old School already described in Park Hill on October 1st, 1878. The other lecturers being as before: Medicine, Dr. Spencer and Dr. Markham Skerritt; Pathological Anatomy, Dr. Spencer and Dr. Markham Skerritt; Surgery, Mr. Tibbits and Mr. Dobson; Midwifery, Dr. J. G. Swayne and Dr. Aust Lawrence; Forensic Medicine and Toxicology, Dr. R. Eager and Mr. W. W. Stoddart; Materia Medica and Therapeutics, Dr. J. E. Shaw; Chemistry, Mr. Thomas Coomber; Botany, Mr. Leipner. (Mr. Coë had just resigned from Surgery, and Mr. Dobson and Dr. Waldo from Anatomy.) This was the last year of the old Medical School in Tyndall's Park Hill, where instruction had been well given by a self-governing body of lecturers since 1833, forty-six years. Their classes were recognised by the examining bodies, and a great many students had been taught there.

Mr. Lister had come to King's College Hospital, then in Lincoln's Inn Fields, in October, 1877, bringing his own house surgeon, Watson Cheyne, and dressers from Edinburgh, and having wards to himself. Despite his writings and the fact that the two most popular text-books in surgery then being used (T. Holmes, 1875, and Erichsen, 1877) recognised his methods of dressing, very few surgeons in London had adopted them, even in a modified form.

I had recently been House Surgeon in King's College Hospital, in charge of all the beds. Sir William Ferguson chiefly used water dressings, but John Wood was using carbolic acid lotion and oil with some measure of success. But our cases rarely healed by first intention—suppuration seemed to be a necessary sequel to almost any wound, and in the wards there was usually some type or other of traumatic fever, erysipelas, or pyæmia, and many deaths. I was at Lister's first lecture, which showed some of the experiments and dealt with the principles upon which he based his treatment of wounds, by preventing the access to them of the living organisms which were floating in the air and in constant contact with the patient. I followed his teaching most attentively, and for a year had the great advantage of watching his practice. It was at once obvious that under the antiseptic treatment as taught and applied by him healing was rapid, and that there was no pain. Suppuration and all kinds of surgical fever were practically never seen, and for me a new outlook in the healing of wounds and in the theory and practice of surgery had begun. When, therefore, I came to the Royal Infirmary in 1878 I naturally brought with me the carbolic steam spray and lotions, the eight layers of antiseptic gauze and boracic lint, etc., the drainage tubes and absorbent ligatures as they were being used at that time in King's College Hospital, and helped more firmly to establish up to date the theory and practice of Lister, which I found was already recognised by Mr. Tibbits and other surgeons in Bristol.[1]

Unhappily, on November 22nd, 1878, Mr. R. W. Tibbits, a very energetic modern surgeon, one of the most active reformers at the Medical School, and at the Infirmary, died suddenly at 36 years of age, and thus failed to go on to the new quarters, under the improved conditions that he had done so much to bring about.[2] A few weeks afterwards Mr. Steele resigned. Thus there were two vacancies for surgeons, which were filled up early in 1879 by the appointments of Mr. Greig Smith, Senior Medical Officer of the Infirmary, and Mr. Harsant, Senior Medical Officer of the General Hospital. These two became very prominent members of the Medical Faculty. Mr. Harsant, Demonstrator of Anatomy for eight years, afterwards held the Lectureship until the appointment of Professor Fawcett, and was very active in all schemes for improving the School.

Greig Smith became a very distinguished surgeon and teacher of surgery, but at the commencement of his career he also much helped the new College and the Medical School by his literary ability. With his assistance the Bristol Medico-Chirurgical Society (founded in 1874) had in 1878 published a volume of *Transactions* of its past work, and in 1880 the Faculty of the Infirmary published a volume of *Reports*, for which he was also largely responsible.

When the time came that these two publications should have issued further volumes it was decided "that the various medical and surgical interests of the West of England ought to be united by the publication of a periodical journal which might appear under the auspices of the Bristol Medico-Chirurgical Society," and in July, 1883, the first copy of the journal of the Society appeared, edited by Greig Smith and published

[1] *Bris. Med.-Chir. J.*, xviii., 201.

[2] "History of the Infirmary," see *Brit. M. J.*, January 11th, 1879, p. 360.

by J. W. Arrowsmith. L. M. Griffiths became Assistant Editor in 1886.

In connection with this journal a large amount of medical literature was collected, many books were sent for review, and later in association with it a library was formed, of which more anon.

On March 19th, 1879, the affiliation of the Medical School with the University College was formally sealed, and this was duly reported to the College of Surgeons, " that an agreement has been entered into by which the management of the School has been put under a ' Governing Body.' The Faculty to be the ' Medical Educational Board.' The ' Governing Body ' will now make application to the ' Council of the University College ' of Bristol to provide without delay suitable buildings and appliances. Future communications to be addressed to ' The Governing Body.' "

On May 31st the following resolution from the College of Surgeons of May 8th was reported :—

" That as recommended by the Court of Examiners, the recognition by the College of the Bristol Medical School be continued, on the understanding that when the new buildings and appliances for the School have been provided, a further report on the subject be furnished to this Council."

On the same day Mr. Coë, the representative on the Faculty reported that the Council of the University College had resolved to commence a portion of its permanent building, and to erect temporary quarters for the Medical School. The Faculty unanimously asked that provision should first be made for the Anatomical Department. It was hoped that lectures in the new buildings might be commenced on October 6th, 1879.

The Syllabus of the Medical School was included with that of the University College, Bristol, in 1879.

GOVERNING BODY OF THE MEDICAL SCHOOL.

Appointed by University College : W. Proctor Baker, Esq. ; F. N. Budd, Esq. ; Right Hon. Lewis Fry, P.C. ; Rev. Professor Jowett ; Dr. Caldicott. Appointed by the Bristol Infirmary : Committee, Rev. G. Heyworth (*Chairman*) ; Staff, Dr. E. Long Fox, Dr. F. Brittan, Dr. Augustin Prichard. Appointed by the General Hospital : Committee, Henry Nash, Esq. (*Chairman*) ; Staff, Dr. Burder, Dr. Siddall, Dr. F. Poole Lansdown. Appointed by the Bristol Medical School : Dr. Shingleton Smith, Dr. Markham Skerritt.

**A.** The Medical School, opened 1879. The first of the University College Buildings on the present site.

**B.** University College Buildings constructed in 1880.

**C.** University College Buildings constructed in 1882.

**D.** The Medical Wing opened in 1892.

Early University College Buildings taken from Architect's Plan dated Feb. 25th, 1890.

On October 1st, 1879, the work of the Medical School under its new Governing Body, with the lecturers mentioned, was transferred to the new brick building in Tyndall's Park, which still remains a part of the Anatomical or Dental Department of the Medical wing of the University of Bristol. (A *on plan, p.* 99.)

That the University College authorities should have given the first part of their new building to the Medical School shows, among other things, their anxiety to do everything possible to help this department, despite their difficulties in coming to a satisfactory arrangement with the Medical Faculty. And it is a pity that generous and combined common effort was not more successful from the start. On the other hand, some members of the Faculty may have been justified in thinking it better to maintain independence, and may have felt uncertain as to the success of the College itself.

Mr. Coë was particularly active in maintaining the rights and claims of the Medical School. He was a strong man and expressed his views strongly.

The position of the Lecturer on Chemistry was also a difficulty, and it is notable that he, who was also the head of the Trade and Mining School, soon afterwards became very active in stimulating the support of the Merchant Venturers in developing that School until it grew into "The Merchant Venturers' Technical College," and was for several years in competition (perhaps even in antagonism) with the University College.

In the Session 1880–1881, while the classes for Chemistry, Engineering and Physics with Physiology remained in the temporary premises of the College in Park Row, all the other College classes were transferred to the new building in Tyndall's Park. (B, C *on plan, p.* 99.)

The Council of the University College now numbered twenty. Besides those mentioned (1876) were Mr. Albert Fry, Mr. John Lysaght, Mr. J. Addington Symonds and Mr. W. Mills Baker, and two representatives of the Medical School.

There were seven Professors: Political Economy, Dr. Alfred Marshall and Mrs. Mary Paley Marshall; Mathematics and Engineering, Dr. Main and Dr. Hele Shaw; Geology, Dr. Sollas.

The next change was the appointment of William Ramsay as Professor of Chemistry, with Mr. Orme Masson as Demonstrator.

On May 3rd, 1880, Professor Lister came to Bristol and distributed the prizes to the medical students in the Theatre of the Museum and Library. He also demonstrated at the Infirmary and at the General Hospital his method of antiseptic operating and the dressings used.

On June 28th, 1881, the prizes were distributed by the Bishop of Gloucester and Bristol, at the Museum and Library, before a large number of visitors, and in 1883 by Dr. Beddoe, F.R.S., and in 1884 by Mr. Augustin Prichard.

The Medical School was fortunate in securing a distinguished doctor or layman to present the prizes. By this annual function, through the courtesy of the city newspapers, attention was drawn to the School, and also to the University College. Moreover, the future University of Bristol was frequently foreshadowed.

The volumes of *The Bristol Medico-Chirurgical Journal* contain reports of these annual prize-givings, and also of the annual dinners of the Colston Research Society.

On November 4th, 1881, the Council of the University College provided additional accommodation for the Physiological Department and £100 for apparatus. Dr. George Munro Smith was recommended as Honorary Demonstrator.

Fenton Evans had been appointed the first Medical Tutor on September 24th, and a part of the Botanical Garden was started by Mr. Leipner on the new University Ground in 1881.

The Secretaries of the Bristol Medical School were: G. D. Fripp, 1832–1840; W. B. Carpenter, 1840–1844; Augustin Prichard, 1844–1854; Dr. Stanton, 1854–1856; Dr. J. G. Swayne, 1856–1858; D. H. Fripp, 1858–1861; Dr. Burder, 1861–1879; Dr. Markham Skerritt, 1879–1883.

In 1883 the post of Secretary was abolished, and Dr. Markham Skerritt became Dean. He continued to be also Chairman of the Medical Faculty until September 22nd, 1905, when he resigned the two posts, as well as that of Professor of Medicine.

On May 2nd, 1884, a scheme for incorporation with University College was approved at a meeting of the Medical Faculty. It was adopted by the Governing Body and forwarded to the Chairman of Council of the University College. When it reached the Council on June 6th legal opinion advised that an agreement entered into by the Council would not be binding on the Governors of the University College. The proposed scheme was therefore withdrawn for the time.

Dr. Lloyd Morgan was appointed Lecturer on Comparative Anatomy in 1884.

At a special meeting held on January 14th, 1887, the Chairman of the Council was hopeful that a fund in support of the College might be raised in conjunction with a Jubilee Scheme.

On April 1st, 1887, the Faculty heartily supported the efforts of the College in their appeal for funds.

On July 1st, 1887, W. H. Harsant was appointed Lecturer on Anatomy, with W. J. Penny as Demonstrator, Dr. Shingleton Smith on Pathology and Morbid Anatomy, George Munro Smith on Physiology, with J. Michell Clarke as Assistant Lecturer.

On December 7th, 1888, the Faculty examined Mr. Hanson's plans for the new Medical School.

On January 3rd, 1890, the Governing Body recommended F. Cook Parsons as Lecturer on Dental Surgery and W. R. Ackland as Lecturer on Dental Mechanics.

On May 17th, 1892, a resolution was proposed by Dr. Shingleton Smith, seconded by Dr. Markham Skerritt, that "The Faculty of the Bristol Medical School approves of the principle of the incorporation of the Medical School with University College, Bristol, and is prepared to join with the Council of the College in the consideration of the subject."

A Sub-Committee—Mr. Dobson, Mr. Greig Smith, Dr. Shingleton Smith and Dr. Markham Skerritt—was appointed. And on June 3rd Mr. Albert Fry, Mr. Lewis Fry, Mr. Schacht, Mr. Worsley and Professor Lloyd Morgan were appointed for the Council; Mr. Arrowsmith and Mr. Schacht to confer with the Faculty as a Building Sub-Committee, and a resolution was passed on July 22nd, 1892, in favour of recommending incorporation.

The Faculty asked that in recognition of his past services to the School, Mr. Coomber, if practicable, be appointed a Professor of Chemistry without a seat on the Senate.

It was, however, resolved that "the Chair of Chemistry in the Medical School be not made professorial inasmuch as the Chair lapses to the University College on its first vacancy, and the subject of Chemistry is now preliminary to the regular curriculum."

On November 18th, 1892, a public meeting was held, when Sir Andrew Clark, President of the Royal College of Physicians, formally opened the new Medical Wing of the University Building in Tyndall's Park, and gave an address. (D *on plan, p.* 99.) In the evening he presided at the Annual Medical School Dinner, being supported also by Lecturers and Governors of the College, and other prominent citizens and old students.

An account of the proceedings and a description of the new building is found in the *Bristol Medico-Chirurgical Journal*, vol. x., p. 277, together with a complete list of Lecturers of the Medical School up to date.

At a special meeting of the Governing Body of the University College on March 28th, 1893, a full incorporation of the Medical School with the College was unanimously approved, and on April 12th, 1893, the agreement was confirmed and sealed.

Thus the Bristol Medical School ceased to have an independent existence and became merged in the University College as its Faculty of Medicine. The fees for lecturers and professors were paid directly to the College, but those received for clinical work done at the hospitals were still kept separate, and did not pass through the College accounts until 1922.

On June 2nd, 1893, the first meeting of the University College Faculty of Medicine was held, sixteen members being present; Dr. J. G. Swayne, Chairman; E. Markham Skerritt, Dean. Professors Dobson, Shingleton Smith and E. Markham Skerritt were appointed representatives of the Faculty upon the Council of the University College.

Certain applications were made for the Professorship of Anatomy, and it was decided that the successful candidate should undertake to devote his whole time to the teaching of Anatomy.

On July 7th it was reported that the Council of the University College had appointed Dr. E. Fawcett, Demonstrator of Anatomy in the Yorkshire College, Leeds, on the recommendation of the Faculty, and J. Paul Bush and C. A. Morton were made joint Demonstrators. On July 28th, 1893, on Mr. Dobson resigning the half Chair of Surgery, Greig Smith was recommended as the sole Professor of Surgery, and in December, 1894, Dr. Aust Lawrence was appointed sole Professor of Midwifery, on the resignation of Dr. J. G. Swayne. In that autumn Greig Smith delivered the address in Surgery at the annual meeting of the British Medical Association, very eloquent and full of knowledge, with great credit to himself and the Bristol School.

On May 28th, 1897, came a most serious loss to the Medical School, Infirmary and the public in the death of Professor Greig Smith.[1]

Dr. James Swain and Mr. C. A. Morton were appointed Joint Professors of Surgery.

In 1898 A. W. Prichard resigned the Chair of Practical Surgery, which he had held for twenty-one years.[2]

On April 7th, 1899, on the resignation of Munro Smith from the Chair of Physiology, it was unanimously recommended that a Professor of Physiology should be chosen who would devote his whole time to the duties of the appointment.

On July 11th, 1899, the Council appointed A. F. Stanley Kent, M.A., Oxon., Professor of Physiology. An arrangement was made so that the whole of the clinical practice of the Infirmary and Hospital should be combined and thrown open to all students. Application was received from the Children's Hospital to be recognised as a place of instruction in diseases of children. A similar proposal was likely to be made from the Eye Hospital and Eye Dispensary.

[1] His biography will be found in *The Bris. Med.-Chir. J.*, xv., 105.

[2] See his reminiscences, *Bris. Med.-Chir. J.*, xviii., 194, which continues those of his father found in vol. x.

On January 5th, 1900, the Dean reported that F. R. Cross would be prepared to become a candidate for the Lectureship in Ophthalmology, in case such a Lectureship be established.

It was resolved unanimously that the Council be informed that in the opinion of the Faculty it is desirable that a Lectureship in Ophthalmology be established.

On February 2nd, 1900, the Dean reported that the Committee of the Joint Faculties were in favour of the admission of the Children's Hospital and the Eye Hospital to the clinical scheme.

A letter was read from Mr. Paul Bush applying for leave of absence on his appointment as Senior Surgeon to the Princess Christian Hospital for the troops in South Africa.

On March 2nd the Dean reported that in accordance with the recommendations of the Faculty the Council had appointed Professor Stanley Kent Lecturer on Bacteriology, and granted leave to him to hold the post of Bacteriologist to the Royal Infirmary, provided it did not interfere with his work at the College. They had established a Lectureship in Ophthalmology and appointed F. R. Cross, and had granted leave of absence to Mr. Paul Bush provided Mr. Munro Smith acted as his substitute.

On April 12th a proposal of Mr. Cross to make his lectures postgraduate and to deliver them at the Eye Hospital was approved.

On September 27th, 1901, occurred the death of Dr. Aust Lawrence.[1]

On March 7th, 1902, Dr. Walter Swayne was appointed Professor and Mr. D. C. Rayner Lecturer in Obstetrics.

On September 22nd, 1905, Dr. Markham Skerritt resigned the post of Joint Professor of Medicine and of Dean and Chairman of the Faculty. He had been the most active constant and capable officer of the Medical Faculty since and even before the foundation of the University College. For twenty-six years he was very rarely absent from a meeting, and kept the records with scrupulous accuracy and fullness. He was made Emeritus Professor of Medicine. He died April 29th, 1907.[2]

On October 3rd it was decided that the posts of Chairman and Dean should be separated, each one to be elected for a period of three years and to be eligible for re-election.

Dr. Michell Clarke was elected Chairman and Professor Fawcett as Dean.

[1] *Bris. Med.-Chir. J.*, xix., 193. [2] *Ibid.*, xxv., 97.

Chairmen: Dr. Walter Swayne, 1911; Mr. Poole Lansdown, 1912; Dr. D. S. Davies, 1914; Dr. Cyril Walker, 1916.

In 1918 Professor Fawcett, F.R.S., was elected to both offices, which he still retains, the offices of Chairman and Dean being again combined. His reputation and the great interest he takes in everything relating to the University and its medical department are of the greatest value.

It was resolved that there should be eight Professors, two in Medicine and two in Surgery, one each in Anatomy, Physiology, Midwifery and one in Pathology (a new Chair).

On October 20th a lecturership on "Diseases of the Nose and Throat" was instituted, to which Dr. Watson Williams was appointed.

On December 1st, in regard to the advertisement for a Professor of Pathology, it was proposed to issue it at the same time as that of Pathologist at the Royal Infirmary, but to await the election at the Infirmary before appointing the Professor at the College.

On February 3rd, 1906, Dr. I. Walker Hall had been elected to both these posts. Dr. Ogilvy and Mr. Prichard advocated the recognition by the Faculty of the Bristol Eye Dispensary as a place for instruction of students: this was acceded to.

On March 2nd, 1906, the Faculty desired that the Memorandum of Agreement, 1893, with the Council of University College should be rescinded, and the Faculty of Medicine to become now an integral part of the College in all respects, and they invited the Council to consider this with them.

A still closer incorporation was thus arranged, but even then the clinical teaching at the hospitals and the associated fees were controlled independently by the hospital staffs; until in 1922 full control of the clinical studies and the fees passed completely into the hands of the University.

On July 5th a report was presented from Council as to the admission of women to the classes for study at the Infirmary and Hospital, and they were now admitted to full medical curriculum of the College on the same footing as men.[1]

On December 16th, 1907, Mr. Prichard reported that the War Office expected that Bristol would form a Field Ambulance. The Director-General expected Bristol to form one Field Ambulance and one General Hospital. The Faculty constituted itself into a Committee and drew up a list of names and ranks

[1] *Bris. Med.-Chir. J.*, xxv., 288.

to be the staff of such General Hospital in time of peace, with Mr. J. Paul Bush as Administrator, and also a list for time of war.

On January 13th, 1908, it was reported that at a meeting of medical men the Faculty of Medicine had been appointed as a Committee to nominate officials in connection with the new Territorial Army Scheme.

In March, 1908, a Lectureship in Ophthalmology in addition to the one already existing was constituted by the Council, and Dr. Cyril Walker was appointed to it on June 3rd.

On May 24th, 1909, the University Charter was granted.

Prior to the granting of a Charter for the University it was necessary to obtain a promise of civic support. This question was fully considered, and it was left to Sir Ernest Cook to lay it before the City Council. He made a most thoughtful and well-prepared speech, leaving no part of the case for a University in Bristol untouched.

"For competition with other countries the fullest technical and scientific instruction are needed. Bristol should be put in an equally strong position with other great centres in England and in Scotland and Germany. Education should be given to every capable citizen from the bottom to the top. A University would be a centre for culture and for research, and for the distribution of knowledge to the districts around it."

After discussion the promise of support was willingly given. Later the Bristol City Council decided to support the University by a penny rate—about £7,000 a year.

On July 6th, 1909, an agreement with the Society of the Merchant Venturers was scheduled to the Charter of the University.

There was a meeting of the Court in July, 1909, at which the Council of the University was elected, with the Right Hon. Lewis Fry, P.C., as Chairman.

Professor Lloyd Morgan's name had appeared in the Charter as first Vice-Chancellor of the University of Bristol, but in order to devote more time to his work on Psychology, etc., he felt it incumbent to resign. Resolutions were passed by Senate and Council of high appreciation of his services, and Sir Isambard Owen, Principal of Armstrong College, Newcastle, was appointed Vice-Chancellor. His reputation as a physician and his wide experience in medical administration made his appointment popular to the Medical Faculty. Moreover, the ability he had shown and the wide experience he had gained in London, Wales, and at Newcastle-on-Tyne, in forming schemes for the development and control of these centres of education, gave confidence that he would render the help that would be needed in organising the new University of Bristol, and in drawing up its Statutes, Ordinances and Regulations.

On September 23rd, 1909, the first meeting of the Medical Faculty (or Board) of the University was held. It commenced with the Vice-Chancellor (Sir Isambard Owen) in the chair (temporarily). Professor Fawcett was elected Dean, and Professor Michell Clarke was elected Chairman of the Board, and he then took the chair.

Much time and labour were needed in deciding details for the medical curriculum. Regulations were drawn up for the degrees in Medicine, Surgery and Dentistry. No. 8 states : " For the purpose of these regulations, ' the University ' shall include the Bristol Royal Infirmary, the Bristol General Hospital, the Royal Hospital for Sick Children and Women, the Bristol Eye Hospital, the Bristol Fever Hospital, and the Bristol City and County Mental Hospital."

On March 2nd, 1910, Mr. Lewis Fry, on behalf of two hundred and thirty subscribers, made a presentation to Professor Lloyd Morgan as an appreciation of his reputation as a man of science and his high work as Principal of the University College for so many years.

On October 20th the first congregation for conferring degrees of the University was held at the Victoria Rooms, when the Vice-Chancellor was supported by the Deans of Faculties, the Lord Mayor and Sheriff, and by the Master of the Merchant Venturers, and by a very large company of ladies, and all that was representative of Bristol in its civic, social, commercial and learned bodies, and many distinguished visitors.

On October 21st, 1910, a meeting was held to promote the establishment of the " University Settlement." Sir Oliver Lodge was one of the chief speakers. In the evening the first dinner of the " Association of Alumni " was held under the presidency of Mr. Munro Smith. Sir Oliver Lodge was the guest of the evening.

On November 15th the new Physiology and Chemical Laboratories at the University were officially opened. A religious service was held by the Bishop of Bristol in the Great Hall, and addresses were given. A procession was then formed—Chairman of Council, Mr. Lewis Fry, the Lord Mayor, City Officials and members of the University; they walked round to the entrance of the new buildings, here Mr. Lewis Fry gave three knocks on the door and was admitted by Lord Winterstoke unlocking the doors on the inside, and declared the new wing open. A large reception was held that evening in the new rooms of the building.

On December 7th a service in commemoration of the founding of the University was held in the Cathedral, the Lord Mayor and Corporation attended. The first University Sermon was then preached by the Rev. A. A. David, Head Master of Rugby.

### THE BRISTOL MEDICO-CHIRURGICAL JOURNAL.

This quarterly, published under the auspices of the Bristol Medico-Chirurgical Society, was first produced in 1883 by J. Greig Smith (Editor), assisted by L. M. Griffiths (Publication Secretary). Later a Committee was appointed to help, and met first on January 29th, 1891. From that date till the present time a Committee has continued, and the offices of the *Journal* have been filled as follows :—

*Editors.*—Mr. J. Greig Smith, 1891 to 1892 (March); Dr. R. Shingleton Smith, 1892 to 1912 (December); Dr. P. Watson-Williams, 1913 to 1926 (March); Prof. J. A. Nixon, 1926 (now in office).

*Editorial Assistants.*—L. M. Griffiths, 1891 to 1899 (October); P. Watson-Williams, 1900 to 1912 (December); J. A. Nixon, 1913 to 1926 (March); Carey F. Coombs, 1926 (now in office).

*Editorial Secretaries.*—Bertram Rogers, 1895 to 1898; James Taylor, 1899 to 1909; J. A. Nixon, 1909 to 1912 (December); J. M. F. Brickdale, 1913 to 1921 (June); A. L. Flemming, 1921 (now in office).

Dr. W. A. Smith acted as Editorial Secretary during Dr. Brickdale's absence at the War in 1916.

### MEDICAL LIBRARY.

In 1890 the members of the Bristol Medico-Chirurgical Society decided to form a Library of the books sent for review and of others given or collected. A room was taken in the Literary and Philosophical Club, 28 Berkeley Square, and there a reading room was formally opened by the President of the Society, Mr. S. H. Swayne, on January 5th, 1891. Many gifts in money and books followed. Mr. L. M. Griffiths was Librarian.[1]

When the new Medical Wing of University College was opened in 1892 the Council had provided a large hall for use as a Library, and arrangements were made for housing together here all the important medical libraries of the district—of the College, of the Royal Infirmary and General Hospital, of the Bristol Museum and Library, and also that of the Bristol Medico-Chirurgical Society, which thus, after two and a half years in 28 Berkeley Square, became merged in the Medical Library of the University College.[2] Greig Smith was Chairman of the conjoint Library until his death, May 28th, 1897, and L. M. Griffiths was Librarian until 1898.[3]

In 1902 C. King Rudge became Librarian of the Bristol Medical Library, and was yearly elected until his death on October 24th, 1926.[4]

In 1911 the Library was obliged to vacate the University College building after an occupation of nearly twenty years, and quarters were found in the East Wing of the Bristol Blind Asylum at the top of Park Street, until it was permanently housed as the Medical Library within the University of Bristol.[5]

### PROFESSORS AND LECTURERS OF THE BRISTOL MEDICAL SCHOOL,

*Theory and Practice of Medicine.*—Dr. W. H. Spencer, 1874–1888; Dr. E. Markham Skerritt, 1876 (Professor, 1893–1906); Dr. R. Shingleton Smith, 1888–1895; Professor J. E. Shaw, 1895–1905; Professor F. H. Edgeworth, 1905; Professor J. Michell Clarke, 1906–1918; Professor J. A. Nixon, 1924.

*Pathology and Morbid Anatomy.*—Dr. Shingleton Smith, 1887–1888; Dr. Barclay J. Baron, 1888–1896; Professor J. Michell Clarke, 1896–1906; Professor I. Walker Hall, 1906.

*Descriptive and Surgical Anatomy.*—Mr. F. R. Cross, 1878–1887; Mr. W. H. Harsant, 1887–1893; Professor Edward Fawcett, 1893.

[1] Report of the first Annual Meeting, *Bris. Med.-Chir. J.*, ix., 313, and xxix., 342.

[2] *Bris. Med.-Chir. J.*, xi., 266, and xiv., 374.

[3] *Ibid.*, xvi., 369, and xviii., 97.

[4] *Ibid.*, xliii., 235.

[5] *Ibid.*, xxx., 346.

*Physiology.*—Dr. Shingleton Smith, 1878–1887; Dr. G. Munro Smith, 1887 (Professor, 1893–1898); Professor Stanley Kent, 1899–1918; Professor G. Buckmaster, 1918.

*Practical Physiology.*—Mr. G. F. Atchley, 1878–1892; Dr. J. Michell Clarke, 1892–1896; Dr. F. Edgeworth, 1896–1898; Professor A. F. Stanley Kent, 1899–1909.

*Theory and Practice of Surgery.*—Mr. N. C. Dobson, 1878–1893; Mr. J. Greig Smith, 1888 (Piofessor, 1893–1897); Professor J. Swain, 1897–1921; Professor C. A. Morton, 1897–1926; Professor E. W. Hey Groves, 1922.

*Practical Surgery.*—Mr. W. P. Keall, 1878–1889; Mr. Arthur Prichard, 1878–1897; Mr. R. G. Poole Lansdown, 1899–1923.

*Operative Surgery.*—Mr. W. J. Penny, 1889–1891; Mr. C. F. Pickering, 1891–1898; Mr. J. Paul Bush, 1898–1921.

*Midwifery and Diseases of Women.*—Dr. Joseph G. Swayne, 1845–1895; Dr. E. A. Aust Lawrence, 1879 (Professor, 1895–1902); Professor W. C. Swayne, 1902–1926; Mr. D. C. Rayner (Lecturer, 1902, Professor, 1926).

*Pharmacology and Therapeutics.*—Dr. J. E. Shaw, 1879–1888; Dr. A. B. Prowse, 1888–1904; Dr. O. C. M. Davis, 1905; Dr. Newman Neild, 1905.

*Forensic Medicine and Toxicology.*—Mr. W. W. Stoddart, 1878–1880; Dr. R. Eager, 1879–1913; Dr. A. J. Harrison, 1881–1899; Dr. G. Parker, 1899–1926; Dr. O. C. M. Davis, 1926.

*Public Health.*—Dr. D. S. Davies, 1886–1926; Dr. R. A. Askins, 1926.

*Comparative Anatomy.*—Professor W. J. Sollas, 1879–1884; Professor Lloyd Morgan, 1884–1909.

*Biology.*—Dr. A. Leipner, 1892–1895; Professor C. Lloyd Morgan, 1892–1898; Mr. S. H. Reynolds, 1895–1910.

*Ophthalmology.*—Mr. F. Richardson Cross, 1900–1910 (Special Lecturer, 1910; Reader, 1922); Mr. C. H. Walker, 1908.

*Laryngology, Rhinology and Otology.*—Dr. P. Watson-Williams, 1907–1921; Mr. A. J. M. Wright, 1913.

*Anæsthetics.*—Mr. J. Freeman, 1913–1923; Dr. A. L. Flemming, 1913.

*Dental Surgery.*—Mr. F. Cook Parsons, 1890; Mr. W. R. Ackland, 1893–1923; Mı. L. E. Claremont, 1923.

*Dental Mechanics and Metallurgy.*—Mr. W. R. Ackland, 1890; Mr. W. A. Hoffmann, 1893–1894; Mr. C. A. Hayman, 1894–1918; Mr. J. Clifford Wing, 1919.

*Dental Anatomy and Physiology.*—Professor G. Munro Smith, 1893–1899; Professor F. Stanley Kent, 1899–1908; Mr. E. A. G. Dowling, 1908–1922.

### BIBLIOGRAPHY.

All the earlier records are generally based on the Bibliographical Memoirs collected by Richard Smith, junior (who died on January 24th, 1843), bound in fifteen volumes, and left by him to the Infirmary, and are still there.

Latimer's *Annals*.

Several volumes of *The Bristol Medico-Chirurgical Journal.*

Vol. xvii., by W. H. Harsant, on " Medical Bristol in the Eighteenth Century."

Vol. xxvi., by J. Paul Bush, " Early History of the Bristol Royal Infirmary."

These two refer to the very early days.

Vol. x., by Augustin Prichard, " The Early History of the Bristol Medical School, from 1833 up till the opening of the University Medical Wing by Sir Andrew Clark in 1892." Contains a full list of the lecturers up to date, with personal notices of many of them.

Vol. xviii., by A. W. Prichard, " Reminiscences of the Bristol Royal Infirmary," continues his father's records, and gives his own personal memories, together with special mention of some of the lecturers.

*A History of the Bristol Royal Infirmary,* by George Munro Smith (J. W. Arrowsmith Ltd.). A very interesting book covering the whole period from 1735 to 1914.

### LISTER—
### REMINISCENCES OF FIFTY YEARS AGO.

Articles were published by Mr. Lister, Professor of Clinical Surgery in the University of Edinburgh, in *The Lancet,* March 13th, 1875, and subsequent numbers on " Recent improvements in the details of Antiseptic Surgery."

The solid organic particles suspended in the air are the causes of putrefaction. Putrescible fluids, such as urine and milk, if no such particles reach them, may be exposed for months to the action of filtered air without undergoing decomposition, and if the fluids which have been previously sterilised are protected by cover glasses, etc., from solid particles falling into them, they will not decompose. Moreover, the materials in water that tend to putrefaction are in the form of suspended particles, not in a state of solution.

Carbolic acid applied in the form of watery solution completely extinguishes the septic energy of putrefaction ferments; infected wounds can be usually rendered aseptic by its use; but the essential in the antiseptic treatment is to prevent putrefaction ever taking place, not to correct it when once established. Deep-seated sepsis and septic sinuses are not always curable by any antiseptic treatment.

The solution of carbolic acid advocated for spray and washing was 1/40, for some cleansing purposes 1/20. The dressing was the antiseptic gauze prepared on Lister's formula, to be changed directly the discharges from the wound threatened to reach the surface, *i.e.* to come in contact with the infected particles outside.

For parts where the gauze was not applicable he advocated solutions of carbolic in olive oil, one-twentieth or even one-tenth soaked into purified lint. Where wounds had already become septic, active efforts should be made to get rid of it. Zinc chloride solution, forty grains to the ounce, was the best application for this purpose.

Lister's attention was drawn to boracic acid (the virtue of which was discovered by Ghan, a chemist in Upsala). He found that boiling water would absorb one-third its weight of boracic acid. From the solution when cold the acid deposited in crystals on lint soaked in it. Lister found this boracic lint, for many purposes, superior to antiseptic gauze, especially when the gauze was difficult to fix, and for operations on the penis, etc.: he also introduced boracic ointment.

Lister himself, by patient personal experiment, worked out the proper strength of his solutions and the details of the composition of his antiseptic dressings. No trouble was too great for him in controlling their proper manufacture. He realised the importance of avoiding tension in wounds, and improved the use of drainage tubes as introduced by Chassaignac. He considered that failure or success of his methods depended on whether they were fully and accurately carried out as taught by him in full detail, and based upon the theories he advocated as to the causation of putrefaction in wounds.

Dr. Clementi, of Caltigirone, in Sicily, in a pamphlet (January, 1875) which takes an extensive survey of the success of Lister's method in Europe, mentions the criticisms which have been offered respecting it, and says: " After having seen Lister's methods employed by surgeons both in Edinburgh and London, I consider that Lister is right when he asserts that if all surgeons do not obtain the same admirable results as himself, it is because they do not accurately carry out the method according to the rules he has laid down."

In England in 1875 some hospital staffs, or individual surgeons, were carrying out his method more or less, but few thoroughly, or with the acceptance of the theory on which it was based. Most were disinclined to follow his practice, but made modifications to suit themselves, using sometimes Lister's own earlier methods, which he had since, in his own judgment, much improved on.

Germany was enthusiastic in his support — France was lukewarm. *The Lancet*, in June, 1875, gave a report of Lister's visits to the German Medical Schools, Munich and Leipsig, in which he was elected an honorary member of the Medical Societies—in the latter place no such compliment had been paid to any British surgeon since Sir William Lawrence. At Halle he was much impressed with Volkman's results on Osteotomy and compound fractures treated in an old hospital, under very unhealthy hygienic conditions. " These results deserve the widest publicity for criticism if they can be challenged in any way; or for imitation if they cannot." Here, also, he saw the great value of Volkman's spoon in cleaning out old septic sinuses, and at once adopted it. He went on to Berlin and Magdeburgh. In all these places he had triumphal receptions by teachers and students.

An important debate took place in the Clinical Society of London on October 22nd, 1875, in which many of the leading surgeons took part. Its report shows how few really worked, in practice, upon the full acceptance of the theory.

Mr. Holmes said he had used the method, and the more he used it the higher did his opinion of it become; but he did not believe in Lister's theory with regard to germs; the general principles were valuable, not the details: without binding himself to his theories, he had no doubt as to the real value of his practice.

Mr. de Morgan said the success of the method was due to the extreme care of the surgeon in his dressings and in general hygienic measures, as seen in Mr. Callendar's cases at Bartholomew's.

Mr. Barwell considered that results were obtained by Lister's method which could not be got otherwise: but in his opinion there were a number of unnecessary details, some of which were repellent and had done the method injury.

Mr. Christopher Heath (an exceptionally fine operator) said he had been astonished at Lister's results which he had seen in Edinburgh. Lister insisted on the minutiæ of his method and regarded them as of great importance; but some of these he found irritating, the technique and details interfered with his eyes and hands.

Mr. Croft said that he had seen Lister's work in Glasgow, and had early adopted it at St. Thomas's Hospital.

Mr. Tom Smith said he accepted Lister's theories and applied them in all their details. He advocated full acceptance of the method, otherwise its rejection: it was not fair to argue on it when only imperfectly or partially applied; but even he was not prepared to say it was very superior to other methods. (He published two lectures in March, 1876, that he had given to the students at St. Bartholomew's Hospital, showing how thoroughly he had studied the subject.)

Most surgeons expressed their belief in the utility of some parts of the method, especially as to scrupulous care and cleanliness of the wound, and the avoidance of all external sources of irritation or putrefaction. But there were others, among them the younger and more scientifically educated, such as Howse (Guy's), Marcus Beck, and Godlee, etc., who were thorough believers, and who strictly followed the practice in detail.

In 1875 also several leading articles appeared in *The Lancet*, written rather perhaps in criticism than in support of the antiseptic method. They advocated the publication of statistics: " It is found that the success of Lister's antiseptic treatment is certainly not greater than that by ordinary methods." " It remains for the advocates of Lister's methods by bringing forward a series of cases to show the superiority of his results over those of others."

Those more scientific observers who were looking on felt no doubt as to what the comparative results would be ; yet statistics are not always reliable and might easily have been made misleading.

In May, 1876, Godlee, at the Harveian Society, exhibited an instrument for the production of a spray cloud used by Lister.

As the full Listerian method with antiseptic gauze became more used—and many of the surgeons in the Royal Infirmary, Edinburgh, were trying it—its expense was found to be a serious objection, and many experiments were tried to replace it by some less complicated dressing, and to find a cheaper method by which suppuration in open wounds might still be avoided—good drainage was looked upon as an essential. (A. F. McGill, Leeds, and others.)

In September, 1876, Lister attended a Medical Congress at Philadelphia, where he was chairman of the surgical section. There was a debate on the treatment of wounds ; the report does not seem to show any wide knowledge of Pasteur's views on fermentation, nor of the antiseptic method as applied by Lister.

Despite Lister's own advocacy of his methods, no distinct conclusion was arrived at as to the relative merits of different systems.

In February, 1877, the death took place of Sir William Fergusson, and it was suggested that Lister should be invited to come to King's College Hospital to succeed him. Mr. John Wood had been appointed to the Chair of Clinical Surgery at King's, and Lister had no desire to give Lectures on Systematic Surgery and he refused the offer of this Chair.

If he came to London it would be in order that he might be able to draw wider attention to the advantages of antiseptic surgery. He wished to be able to demonstrate to a larger circle of surgeons his methods as applied to the actual cases that came for treatment, an opportunity of teaching practical surgery at the bedside, and of demonstrating the antiseptic system as invented by him.

In March the Edinburgh students memorialised Lister to remain with them. In his reply he said: " There seems nothing in London at present good enough to tempt me away from this school," but later he accepted the offer of King's College, where he was appointed an extra Professor of Clinical Surgery, with a special department of wards, house surgeon, staff, etc.

He resigned the Chair of Clinical Surgery in Edinburgh, and at the end of the summer session, July, 1877, he gave a valedictory address to the students and took leave of his Chair. He came to London and gave the introductory address at King's College Hospital in October on " A Record of Experiments on Fermentation." He showed his methods and the apparatus used, and how he had applied the lessons learned, and the principles involved to the prevention of surgical sepsis.

Some remarks that Lister had made to his Edinburgh class on clinical teaching in London had not unnaturally provoked an hostility to him amongst some of the senior surgeons—letters had passed on the subject—but on the whole he was received in London in a generous spirit.

On December 18th, 1877, he gave a remarkably lucid and well-reasoned address on the " Lactic Fermentation and its bearings on Pathology " : the paper was very well received, Dr. Bastian and others spoke on it.

Lister was a very charming personality, very modest but with plenty of strength of purpose—he was not inclined to be combative in his statements in defence of his views, nor aggressive in criticism of those with which he did not agree. He went on unshaken in his confidence of opinions which were based on years of investigation and inquiry, the accuracy of which he had proved to his own satisfaction.

No doubt he was disappointed at the limited number of his active followers in London who at first supported his views, especially as it was in great contrast to the wide support he had received in Edinburgh and abroad.

But his teaching rapidly gained in popularity, especially among the more scientific thinkers ; and as the results of his methods became more widely known, it was obvious that the claims he was making for his system were fully justified.

Criticisms, however, continued to be made, and his early days in London were not easy ones. In his own clinique failures were almost unknown. If one occasionally occurred, the reason could generally be traced to some act of carelessness in detail of treatment.

The method was by no means universally followed, but an influence was produced, even on those who appeared to be his most active opponents.

I remember being shown at times, soon after he came to London, comments in the Press which were made in a very unfair spirit, without giving names, on the risk of some operations which were being done (wiring the patella, opening of joints, etc.), and which suggested the prosecution of the surgeon in case of a fatal result. Criticisms of the ill-doings of the bacteria in surgical wards and theatres were treated in a spirit of banter and levity which betrayed ignorance, and showed a narrow and inaccurate view which may now be well forgotten.

His teaching was mainly directed to the healing process in ulcer and abscess, and in injuries whether open or protected by the skin, and to the prevention of sepsis and surgical fevers.

He was a very painstaking and successful operator with original ideas, and his cases all did well.

He was handicapped in consultation practice because he could not recommend certain serious operations unless he himself or a very limited number of his pupils kept charge afterwards. The doctor could not always be relied on to continue the antiseptic treatment of the case. He did not feel justified in running any risk of suppuration where it might be disastrous. But he was fully relied on to do his best, both for his colleague and his patient.

His teaching was not of the tutorial type, nor altogether adapted to the ordinary examination purposes of the day—some of the examiners did not accept his views in detail, and from that point of view classes at some other hospitals may have seemed more useful for students.

Sometimes we hear of " aseptic " as against " antiseptic " treatment, but surely the one is a natural corollary of the other. Lister's aim was aseptic to prevent sepsis taking place. Modern scientific surgical treatment, whether antiseptic or aseptic, is due to the work, the genius, and the initiative of Lister.

F. R. CROSS.

## Figures and Tables

**Figure f1:**